# Experimental disturbance and productivity gradients drive community diversity in aquatic mesocosms

**DOI:** 10.1002/ece3.10049

**Published:** 2023-05-07

**Authors:** Jacob D. Hart, David G. Jenkins

**Affiliations:** ^1^ Department of Biology University of Central Florida Orlando Florida USA

**Keywords:** aquatic vegetation, dynamic equilibrium model, experiment, intermediate disturbance hypothesis, intermediate productivity hypothesis, mesocosm

## Abstract

Combined effects of disturbance and productivity on ecological diversity have been considered for decades as the dynamic equilibrium model (DEM) but are rarely tested together. Instead, most studies focus on either the intermediate disturbance hypothesis or sometimes the intermediate productivity hypothesis. In addition, most analyses of disturbance and productivity effects have relied on nonexperimental patterns, limited sample sizes, inaccurate proxies for productivity, and/or simple measures of diversity. The DEM operates at regional and local scales; here, we conducted a year‐long experiment at local scales using submersed aquatic vegetation in outdoor mesocosms with a factorial combination of physical disturbance and productivity treatments. We evaluated diversity in several ways, directly measured productivity, and compared alternative hypotheses using model selection. The DEM was supported for effective diversity; both productivity and disturbance effects were clear, though productivity effects were stronger. Other diversity measures for the simple communities in the mesocosms did not clearly reflect treatments. The DEM is a valuable general framework for understanding disturbance and productivity effects on ecological systems and is made more general by minor conceptual adjustments here.

## Graphical abstract

As predicted by Huston's dynamic equilibrium model (DEM), both disturbance and productivity affected the diversity of aquatic vegetation in outdoor mesocosms during a 1‐year experiment. The DEM has been rarely tested experimentally but brings clarity to understanding processes that affect biological diversity.

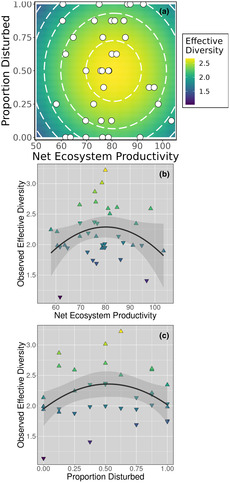

## 1 INTRODUCTION

**FIGURE 1:**
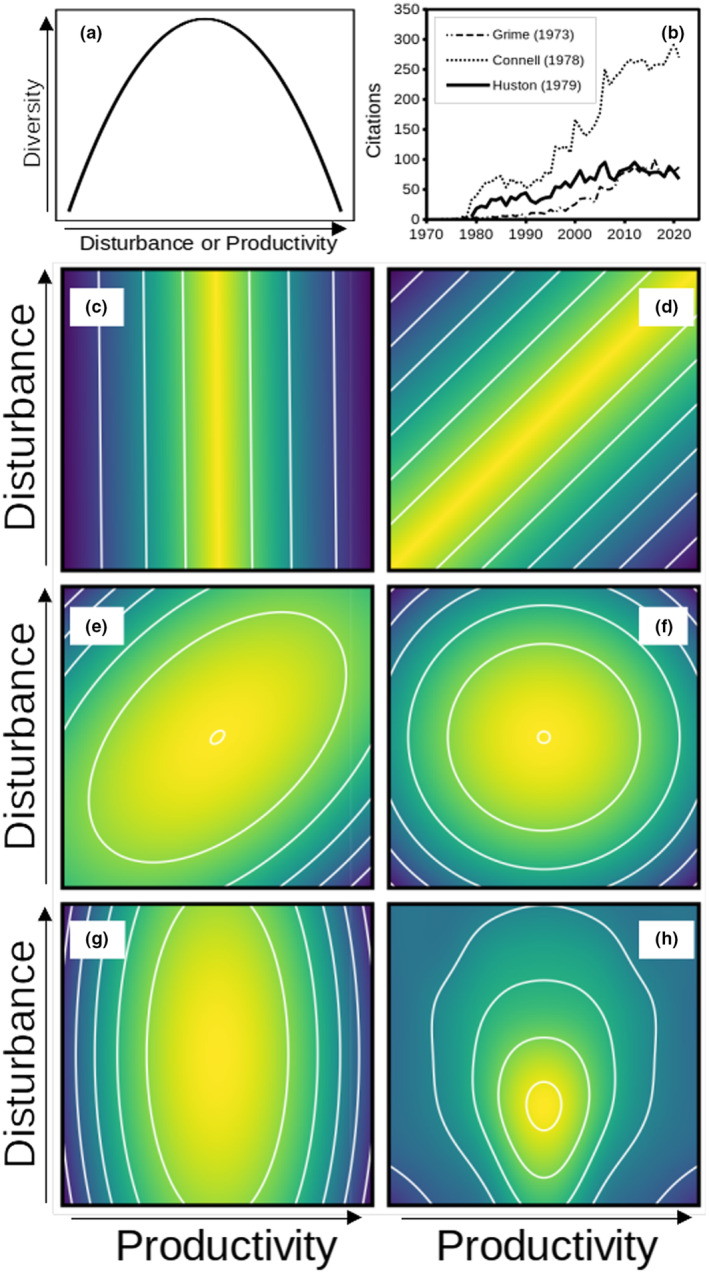
Alternative conceptual views of the intermediate disturbance and intermediate productivity hypotheses (IDH and IPH, respectively) and their additive combination (the dynamic equilibrium hypothesis; DEM). Warmer colors indicate greater diversity. (a) The univariate IDH or IPH expects an arch‐shaped peak of diversity along an intermediate disturbance or productivity gradient. The arch is modeled most simply by a quadratic equation. (b) The IDH (Connell, 1978) remains cited most often, whereas the IPH (Grime, 1973) and the more complex DEM (Huston, 1979) are cited less often. Data from Web of Knowledge, obtained March 9, 2023. (c) The partial view of the IDH (or here the IPH, which ignores the second driver, disturbance) translates to vertically‐oriented contours with elevation as diversity. (d) In landscapes that include patches with different productivity, the effect of local disturbance is reflected as a contour “ridge,” where a gradient at any transect would reveal only part of the story. (e) At local scales, a contour “hill” is expected. Figures (d) and (e) are based on Huston (2014). Alternative local effects may include: (f) dual and symmetrical effects; (g) dual effects but one driver dominates; or (h) fine‐tuned contours more complex than simple quadratic equations. Axes in (c–h) could be transposed but are arranged as in Huston (2014).

Disturbance and productivity are each commonly expected to affect biodiversity, but the details of those relationships are complex, vary among systems, and have long been debated (Adler et al., 2011; Fox, 2013; Huston, 2014; Mackey & Currie, 2001). Each driver was hypothesized to act alone, leading to the well‐known intermediate disturbance and intermediate productivity hypotheses (IDH and IPH, respectively; Al‐Mufti et al., 1977; Connell, 1978; Grime, 1973; Huston, 2014). Each hypothesis expects a unimodal response of diversity among places arrayed in a disturbance or productivity gradient (Figure 1a). The IDH assumes a general competition‐colonization tradeoff and expects low biodiversity due to competitive exclusion when disturbance is infrequent or weak, and due to mortality when disturbance is frequent or intense (Connell, 1978; Grime, 1973). Greatest biodiversity then occurs in places with intermediate disturbance frequency or intensity.

The IPH is also based on competition and mortality but caused by productivity (Grime, 1973; Huston, 1979, 1994, 2014) where low productivity causes low biodiversity because fewer resources exist, but high productivity causes competitive exclusion, such as when algal blooms reduce aquatic macrophytes (Figure 1a). The IPH has attracted less attention than the IDH (Figure 1b), perhaps in part because it was rarely treated as a distinct hypothesis or even named until ~20 years later (Lawton, 1995). For example, a large study (Adler et al., 2011) on the generality of the IPH for species richness cited Grime (1973) and explained the hypothesis without naming it.

The IDH and IPH were joined early in the dynamic equilibrium model (DEM; Huston, 1979, 1994, 2014) to recognize that both disturbance and productivity affect diversity in a landscape, where one effect may confuse evidence for the other unless both are evaluated. Thus the IDH and IPH are subsets of the DEM, and the more complex DEM hypothesis has also lagged behind the IDH in attention or debate (Figure 1b). The expected DEM pattern was depicted by Huston (1979), with the hump‐shaped pattern classically expected from IDH or IPH viewed from above and diversity depicted as contours (Figure 1c; here using the IPH without disturbance effects). Where both disturbance and productivity affect diversity, Huston (1979) depicted the DEM as reflecting disturbance and productivity gradients exist across a landscape to “tilt” the Figure 1c contour to represent both effects (Figure 1d; based on Huston, 1979). The dual effects of disturbance and productivity thus lead to varied diversity on either axis (contour lines in Figure 1d) and help to explain confusion for either the IDH or IPH (Huston, 2014). We also note that contour ridges in Figure 1c,d are symmetrical but need not be.

So far, we summarized the DEM for regional diversity, where curves or contours represent landscapes with different productivities and include local patches with different disturbance levels (Huston, 1994, 2014). If instead one considers local scales, where both the IDH and IPH act on distinct locations, then the two hump‐shaped drivers act additively on local diversity. As a result, the angled regional “ridge” among locations in Figure 1d becomes a “hill” (Figure 1e; Huston, 1994, 2014).

The above graphical summary of the DEM (based on Huston, 1979, 1994, 2014) indicates three malleable features of a DEM contour plot (beyond axis ranges): the scale of the contour pattern (i.e., landscape ridge or local hill), its shape (e.g., symmetry or skewness), and its orientation in the disturbance‐productivity plane. Here we set aside landscape‐based contours (ridges; Figure 1c,d) to consider hill‐shaped DEM contours for two reasons: additional DEM contours are possible but relatively unexplored, and because we experimentally focus on local diversity, where both disturbance and productivity treatments are applied to replicate mesocosms.

A local contour hill (e.g., Figure 1e) oriented similarly to the regional ridge (Figure 1d) is not a default, though it has been observed (Janousek & Dreitz, 2020; Kassen et al., 2004). Mathematically, a skewed hill may be approximated with intercept terms in additive quadratic equations or by more complex models. More broadly, other DEM contour hill shapes (e.g., Figure 1f–h, and other potential shapes) may arise if diversity responds to disturbance differently than it responds to productivity. In turn, the shape of DEM “hills” should help evaluate the dual local effects of these fundamental drivers of ecological diversity. For example, if additive IDH and IPH effects are each as in Figure 1a and matched, then contours are a symmetrical hill in the DEM plane, without orientation or skew (Figure 1f). If one driver (e.g., productivity) clearly dominates, but both curves are shaped as in Figure 1a, then a “stretched” hill is obtained (Figure 1g), oriented similar to Figure 1c. If the math underlying the Figure 1g pattern is finely tuned, then the pattern may be skewed similar to Figure 1e but without a dual orientation (Figure 1h).

From these simple examples, it should be apparent that DEM contour plots can help understand the relative effects of disturbance and productivity on biodiversity (Huston, 1979, 1994, 2014). Moreover, we infer that orientation in the DEM plot is caused by relative differences between IDH and IPH effects, and that skew in the curve requires a narrow set of coefficients or added terms. The above expectations assume that other potential factors (e.g., site history) do not modify IDH or IPH effects, which is more likely the case in simple or controlled experimental systems (e.g., Kassen et al., 2004) than in natural systems.

One might expect that empirical evidence for the DEM summarized above has already accrued. It has not (see Figure 1b), perhaps related to the greater complexity of the DEM and relative difficulty in simultaneously observing full, orthogonal disturbance and productivity gradients in nature; it can be hard enough to evaluate either the IDH or IPH in nature, let alone both (Huston, 2014; Wilkinson, 1999). But four other problems also contribute to confusion, especially surrounding the IDH beyond its well‐known debates (Fox, 2013; Huston, 2014). Work here sought to address these problems.

First, low sample sizes have made it difficult to detect effects. Almost 2/3 (64%) of IDH studies gathered in a large review (Mackey & Currie, 2001) lacked sufficient sample size to discern between a hump‐shaped and a linear curve (Jenkins & Quintana‐Ascencio, 2020). As a result, reported failures to find consistent evidence for the IDH (Mackey & Currie, 2001) and resulting criticism (Fox, 2013) may be more related to the conduct of tests than the idea itself.

In addition, empirical experiments to test the DEM have been surprisingly rare. For example, 58% of papers citing Huston's (2014) explanation of the DEM (per Google Scholar, 10 March 2023) included the word “experiment.” Of those papers, most simply cited Huston (2014) when considering the IDH and its debate (e.g., Fox, 2013; Huston, 2014), or the IPH. Among the 138 papers with the word “experiment,” we found only one (1.4%) peer‐reviewed paper (Groendahl & Fink, 2017) reporting an empirical experiment to test the DEM at local scales; algal microcosms were used to evaluate interactive effects of consumer richness and productivity on algal biodiversity. Other studies are informative for the IDH, IPH, and DEM but used monitoring data across landscapes (e.g., Janousek & Dreitz, 2020) or accounted for landscape productivity when evaluating disturbance effects (e.g., Chillo et al., 2020). Though surely not an exhaustive sample, the apparent rarity of experimental DEM tests, especially at local scales, indicates that the DEM remains an incompletely tested but general framework for two fundamental drivers of biodiversity—disturbance and productivity.

Two more challenges have burdened the understanding of the IDH, IPH, and DEM. First, studies have often measured diversity as simple species richness, despite known problems with that fundamental measure (e.g., Gotelli & Colwell, 2001; Jost, 2006; Roswell et al., 2021). Secondly, productivity has often been measured using standing stock biomass (e.g., Adler et al., 2011), but biomass is a static measure (e.g., g m^−2^) whereas productivity is a rate (e.g., g m^−2^ year^−1^), and using biomass as a proxy for productivity introduces error (Jenkins, 2015).

As a result of all the challenges outlined above, considerable confusion remains about the DEM and its constituent hypotheses (IDH and IPH). We conducted an experiment to test the DEM using aquatic plants in outdoor mesocosms to obtain strong replication and experimental control of treatments while retaining some realism (Odum, 1984; Petersen & Hastings, 2001). We applied both physical disturbance and nutrient additions in a factorial design to encompass wide ranges of both drivers and evaluate results relative to theoretical expectations (Figure 1). We emphasized effective diversity (^1^ *D* = *e* ^*H*'^, where *H*′ is Shannon diversity and the ^1^ *D* notation relates to Hill numbers; Jost, 2006) as the response variable but also evaluated diversity using dry‐mass biomass, taxon richness, and evenness (Jost, 2010). We quantitatively estimated both disturbance and production as continuous variables. And we directly measured production rather than using proxies such as categorical nutrient levels (e.g., Huston, 1979; Kneitel & Chase, 2004; Korpinen et al., 2007; Scholes et al., 2005) or biomass (e.g., Adler et al., 2011; Laliberté et al., 2013; Pollock et al., 1998) that can introduce errors because proxies are not linear equivalents of productivity (Jenkins, 2015). Finally, we compared alternative models representing alternative hypotheses and related results to theoretical expectations for the DEM (Figure 1) to determine which relationships between productivity, disturbance, and diversity were experimentally supported.

We expected that the wide range of experimentally‐applied disturbance and production levels would both affect macrophyte diversity, as intended. We could not predict the precise shape or orientation of the DEM plot beyond an elliptical or rounded shape (e.g., Figure 1e–h), though any of those would support the DEM. Potential evidence against the DEM would be results similar to Figure 1c that support the IPH or IDH despite both productivity and disturbance treatments, or a lack of diversity response (a null effect). Based on well‐known eutrophication effects (e.g., Smith & Schindler, 2009), we also expected algae would become more prominent with more nutrients and perhaps more disturbance (as a result of disrupted macrophytes). We also expected macrophytes would differ in vulnerability to the mechanical disturbance applied here due to some species' capability to reproduce from fragments.

## 2 MATERIALS AND METHODS

### 2.1 Experimental design and data collection

**FIGURE 2:**
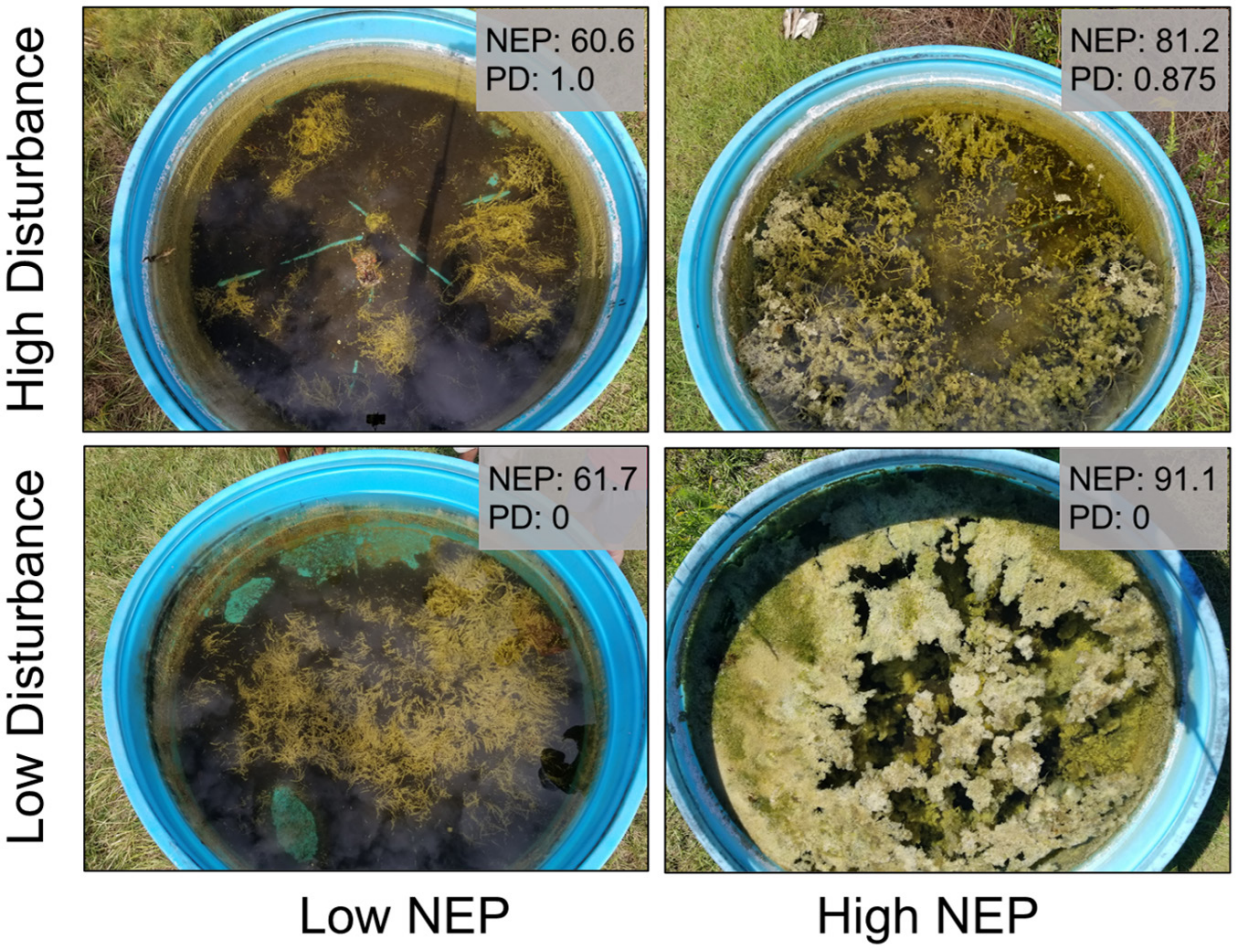
Representative mesocosms arranged by relative levels of net ecosystem production (NEP; cumulative net mg DO L^−1^) and proportional disturbance (PD; % of area raked). Boxes contain measurements for these values. Extremes are shown here for illustrative purposes, though treatments covered effective gradients of both variables. The images presented here may be considered broadly representative; replicates with low NEP and low disturbance regimes consistently grew short, sparse *Chara*, and little else (bottom left). Mesocosms with low disturbance and high production were dominated by filamentous algae (bottom right), whereas those that were highly disturbed with low production exhibited sparse patches of *Chara*, with few other species surviving (top left). Replicates with high levels of both production and disturbance exhibited limited growth of filamentous algae and heterogenous structure of macrophyte communities (top right).

The experiment used 40 outdoor mesocosms (2.4 m diameter cattle tanks), where treatment combinations were randomly assigned to an individual mesocosm and maintained throughout the year‐long experiment (October 2017–November 2018; see Figure 2 for representative treatment combinations). It is important to note that production and disturbance treatments were quantitative rather than categorical; a gradient of ecosystem net primary production was caused by fertilizer treatments but quantified (described below), and disturbance was applied in a quantitative gradient. Production and disturbance treatments were applied in a factorial experimental design with two blocks to account for mesocosm locations (i.e., 2 sets of 20). However, blocks never exhibited significant effects in any results and actually interfered with inference on experimental treatments; blocks were omitted from analyses described below.

Mesocosms were started with topsoil and aquatic sediment in a 2:1 ratio to a depth of ~5 cm. Aquatic sediment contained a potential seed bank (17 plant species and macroalgae) from a prior experiment, where saturated sediment had been stored outdoors. Sediment was mixed with shovels, added to mesocosms with topsoil, and mixed again in cattle tanks. Mesocosms were then filled with ~2200 L unchlorinated well water and water depths were maintained thereafter at 30–40 cm depth by rain and additional well water as needed.

Each mesocosm's randomly assigned disturbance level (one of eight) was used for the entire experiment (see “Data Availability Statement” below for details). Disturbance was mechanical, where mesocosms were dredged by hand using a garden rake (48 cm wide, 16 tines, ~3 cm apart) at ~60‐day intervals, and where treatment levels corresponded to the percent of the benthic area raked (0%–96%, in 8 increments). Shallow sediment depths ensured that raking penetrated through to the plastic bottom to disrupt roots and rhizomes. The direction that sediments were raked (from edge to middle or vice versa) was randomized per treatment event per mesocosm to avoid repeatedly piling sediment in portions of the raked area. The percent of benthic area raked remained the same for the entire experiment and was easily repeatable because the radius of a mesocosm was less than the rake handle length, mesocosms were relatively shallow, and different levels of disturbance were a simple matter of counting rake paths per mesocosm. Locations of raking within a tank were randomized for each event, so that any given plant in a mesocosm had a chance of being raked according to the portion of area raked. Raking dislodged or broke vegetation and stirred up sediment to increase turbidity. Raked vegetation remained in mesocosms, which appeared to affect conditions by providing a floating substrate for filamentous algae attachment and by shading intact rooted vegetation.

Each mesocosm was randomly assigned a productivity category (stratified by disturbance levels for a factorial design) for treatment with fertilizer in one of three levels: none; medium (50 μg P L^−1^); and high (100 μg P L^−1^). Phosphorus was the main focus for productivity because freshwater systems are typically P‐limited (Smith & Schindler, 2009), and medium and high levels approximated mesotrophic and eutrophic freshwaters (Carlson & Simpson, 1996). Potassium phosphate was first added in October 2017 at the experiment start, followed in June 2018 by commercial fertilizer (MiracleGro brand, NPK ratio = 24:8:16) according to the proportions above to ensure sufficient nutrients remained available to maintain treatment differences.

Productivity was quantitatively estimated in each mesocosm as net ecosystem production (NEP; mg DO L^−1^), which represented entire mesocosms because vegetation, periphyton, and heterotrophs were submersed (i.e., no emergent vegetation existed). We estimated NEP about monthly using diurnal (sunset, sunrise, and again at sunset) changes in dissolved oxygen (DO) to obtain gross primary production (GPP) and respiration (*R*), where NEP = GPP – *R* (Odum, 1956). Water temperature and mean depth (*z*, from 3 locations) were also recorded per mesocosm and mean wind speed for the interval was obtained from a nearby weather station. These data were used in a modified version of (Odum, 1956), which is robust to the effects of photosynthetic light saturation, afternoon production depression, and fluctuating light levels but sensitive to changes in reaeration (Kosinski, 1984). Reaeration was handled here using nonlinear estimation of a reaeration coefficient (m h^−1^), based on mean wind speed during the interval:

(1) $$k=0.00214\times {\text{wind}}^{2}+0.0002008\times \text{wind}–0.00104155$$

Where that function was fit (*R* ^2^ = .886) to data in table 1 of Wanninkhof et al. (1985). The NEP = GPP – *R* equation can then be modified as:

(2) $$\mathrm{\Delta DO}\;h^{-1}=\mathrm{GPP}–R+\left(k/z\right)d$$

where terms are as defined above and *d* is the saturation deficit, which was calculated as the difference between the equilibrium solubility of oxygen (*C*_S_; mg DO L^−1^) and measured concentration (*C*; mg DO L^−1^). *C*_S_ was calculated as 28.9554–5.71703 × ln(Temperature + 12.0947), based on an equation fit (*R* ^2^ = .99) to data in table 1 of Benson and Krause Jr (1980). Re‐arranging Equation (2) yields:

(3) $$R=\mathrm{GPP}+\left(k/z\right)d–\left(\mathrm{\Delta DO}\;h^{-1}\right)$$

Because GPP = 0 at night (i.e., sunset to sunrise), overnight *R* (mg DO L^−1^ h^−1^) is:

(4) $$R=\left(k/z\right)d–\left(\mathrm{\Delta DO}\;h^{-1}\right)$$

Likewise, daytime (i.e., sunrise to sunset) GPP (mg DO L^−1^ h^−1^) is:

(5) $$\mathrm{GPP}=\mathrm{\Delta DO}\;h^{-1}+R–\left(k/z\right)d$$

Having estimated GPP and *R* with adjustments for winds, NEP was calculated as GPP – *R* (Odum, 1956).

We estimated NEP 17 times through the year (about once every 20 days), where each estimate represented a 24‐h interval. Mesocosms had variable NEP trends through time, but a single production estimate per mesocosm was needed for analyses. Two methods to summarize NEP were compared: mean daily NEP and cumulative annual NEP, which was based on integrated area under a local estimation (loess) function for NEP ~ time. Loess functions fitted data points well, and the two estimates were closely matched, with a nearly 1:1 slope when values were scaled (slope = 0.98), an intercept very near zero (−1.5 × 10^−16^), and a tight linear fit (*R* ^2^ = .95). We chose to use cumulative annual NEP to represent productivity effects and emphasize again that this procedure provided empirical productivity values.

After 1 year, macrophyte communities were assessed visually for percent cover and by biomass. Plants and filamentous algae occupied vertical strata, much like ground, sub‐canopy, and canopy layers in an underwater forest. Similar to vertically‐stratified forest analyses (e.g., Onaindia et al., 2004), we estimated percent cover per taxon (to the nearest 5%) at three heights within mesocosms: bottom, mid‐depth, and water surface. Percent cover is a reliable estimate of abundance for vegetatively‐reproducing macrophytes and macroalgae, especially given the mechanical disturbance (raking) used here. Visual estimation at all three depths was simple in the shallow mesocosms; in some cases, surface vegetation was gently moved aside to better see bottom layers. For each layer, the unvegetated area was also recorded, minimum values were 1% for rare plants, and vegetated area + unvegetated area = 100% of cover.

Mesocosms were then slowly drained by siphoning through hoses and vegetation (i.e., vascular plants and macroalgae (*Chara*, *Nitella*) anchored in sediments) was harvested by hand, separated by taxon, dried (48 h at 105°C), and weighed to the nearest mg. Thus vegetation that was rooted or anchored to the raked substrate was evaluated for biomass; filamentous and planktonic algae in the water column were not evaluated for biomass. However, filamentous algae were included in percent cover (i.e., an important basis for analyses below) and taxonomic richness estimates.

Mean percent cover per taxon per mesocosm was analyzed as the average among vertical layers (surface, middle, bottom). To help understand those results, we also analyzed separately percent cover for each layer and taxon, where we prioritized taxa that occupied the most cover. We quantified plant diversity as effective diversity (^1^ *D*; defined above), calculated for each of the mean percent cover and biomass. We also evaluated taxon richness (^0^ *D*) and evenness per mesocosm but emphasize ^1^ *D* here because simple communities constrained the range of response for richness and evenness.

We compared expected quadratic models for the DEM to alternatives, including linear effects; each of IDH and IPH alone; partial combinations (e.g., the IDH and a linear effect of productivity), and a null (Table 1). All models included continuous predictors of cumulative NEP and disturbance intensity (proportion of area) and were computed as generalized linear models (GLMs), using the gamlss package in R (Rigby & Stasinopoulos, 2005), In the absence of smoothing functions for a generalized additive model (GAM), the gamlss package produces GLMs. An advantage of gamlss is that it makes available 86 potential distribution families (some of which also enable varied link functions) to best meet regression assumptions of homogeneous variance and normally‐distributed residuals. By comparison, glm provides eight families. We used families related to classic options, but this flexibility helped to best meet model assumptions (i.e., homogeneity of variance and normality of residuals), which were evaluated using diagnostic plots in the gamlss package. Alternative models reflecting hypotheses (Table 1) were compared with the Akaike Information Criterion corrected for small sample size (AIC_c_; Burnham & Anderson, 2002), using the bbmle package in R (Bolker & R Development Core Team, 2020) and based primarily on AIC_c_ weights (*w*_*i*_). If models were similar in rank (i.e., δAIC <2, often for nested model sets), we used reason based on AIC‐based model selection via the stepAIC command in the MASS package (Venables & Ripley, 2002) and coefficients significance. This inference process typically retained the more inclusive model as being most efficient among those listed. Adjusted *R* ^2^ values were then used to “critique” outcomes (Bolker, 2008). Scaled (units = 1 standard deviation) coefficient terms provided fair comparisons of relative effect sizes (i.e., model coefficients) of disturbance and productivity on diversity. Finally, we adopted language suggested by Dushoff et al. (2019) regarding statistical significance, where “clear” is preferred to “significant.”

**TABLE 1:** Alternative hypotheses and corresponding univariate models used for effective diversity (^1^ *D*) as a function of productivity, disturbance, or both.

Hypothesis	Model
No detectable effects	Null
Diversity changes linearly with productivity	^1^ *D* ~ productivity
Diversity changes linearly with disturbance	^1^ *D* ~ disturbance
IPH: Diversity changes as a ∩ with productivity	^1^ *D* ~ productivity – productivity^2^
IDH: Diversity changes as a ∩ with disturbance	^1^ *D* ~ disturbance – disturbance^2^
Diversity changes linearly with both productivity and disturbance	^1^ *D* ~ productivity + disturbance
Diversity changes linearly with productivity but as a ∩ with disturbance	^1^ *D* ~ productivity + disturbance – disturbance^2^
Diversity changes as a ∩ with productivity but linearly with disturbance	^1^ *D* ~ productivity – productivity^2^ + disturbance
DEM: Diversity changes as a ∩ with both productivity and disturbance	^1^ *D* ~ productivity – productivity^2^ + disturbance – disturbance^2^

## 3 RESULTS

Results for effective diversity (^1^ *D*) based on mean percent cover were clear and supported the DEM (i.e., the most plausible model included quadratic disturbance and quadratic NEP; Table 2). A model representing the DEM was not clearly separated in AIC rankings (i.e., δAIC_c_ < 2) from the IDH model, which was a nested subset of the more inclusive DEM model. Moreover, the DEM model was irreducible according to AIC‐based model selection, and all terms were significant (Table 3). Based on the above evidence, we inferred that the results supported the DEM model. In the DEM model, production effects were stronger than disturbance effects, as evidenced by greater scaled effect sizes for NEP than those for Disturbance (Table 3). This result appears to conflict with the relatively low rank of the IPH model in AIC‐based model comparisons (Table 2). We inferred that this result further supported the DEM relative to its constituent hypotheses (IPH or IDH) because simultaneous disturbance effects confounded productivity effects in the IPH model. All disturbance and productivity terms in the DEM model were clearly different from zero, based on *p* values (Table 3).

**TABLE 2:** Model comparison results for effective diversity (^1^ *D*) of percent cover data.

Model	AIC_c_	δAIC_c_	Terms	Weight (*w*_*i*_)
IDH	43.6	0.0	4	0.394
DEM	44.6	1.0	6	0.233
IDH + linear production	45.9	2.3	5	0.126
IPH	46.4	2.9	4	0.094
Null	47.0	3.4	2	0.072
IPH + linear disturbance	49.1	5.5	5	0.025
Linear production	49.1	5.5	3	0.025
Linear disturbance	49.3	5.8	3	0.025
Linear disturbance and production	51.6	8.0	4	0.007

*Note*: Alternative models are sorted by AIC_c_ weights (*w*_*i*_), which represent the probability that a listed model is most plausible in the analyzed set. Given δAIC_c_ <2, the IDH and the more inclusive DEM model were similarly plausible; the IDH was a nested subset of the DEM model, which was not reduced by stepAIC and all its terms were significant (see Table 3).

**TABLE 3:** The DEM model for effective diversity (^1^ *D*).

	Estimate	SE	*t* Value	*p* Value
Intercept	2.332	0.059	39.4	<.001
NEP	1.482	0.609	2.4	.02
NEP^2^	−1.458	0.612	−2.4	.02
Disturbance	0.666	0.233	2.8	.007
Disturbance^2^	−0.685	0.228	−3.0	.005

*Note*: Adjusted *R* ^2^ = .29; Weibull distribution Σ = 1.93 + 0.13 (SE); degrees of freedom = 6 & 31. Net ecosystem production (NEP) and disturbance effects were scaled for fair comparisons of effect sizes (units = standard deviations). Hump‐shaped effects for NEP and disturbance are indicated by signs on the coefficients.

**FIGURE 3:**
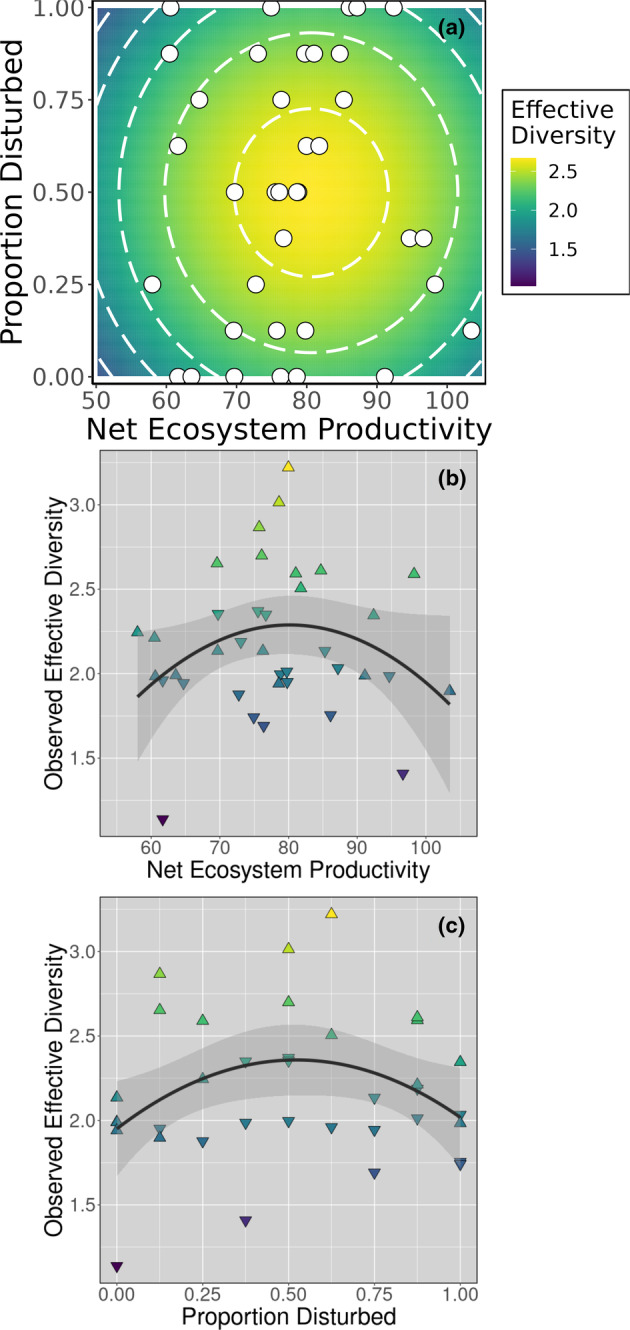
Predicted effective diversity (^1^ *D*) per the most plausible model (see Table 3 for model details). (a) Predicted values depicted as a response surface to compare with hypothetical surfaces of Figure 1. White circles represent combinations of productivity and disturbance obtained in experimental mesocosms. Effective diversity (^1^ *D*) is also depicted as a function of (b) net ecosystem productivity or (c) proportion disturbed, where triangle colors match the legend and direction indicates whether values were above or below the predicted surface in (a). Curves in (b) and (c) include 95% confidence intervals and were fitted by the generalized linear model to represent classic tests of productivity or disturbance alone (i.e., without accounting for the combined effects of both experimental treatments). Net ecosystem productivity units = cumulative net mg DO L^−1^ (see Section 2 for details).

The model representing the DEM was modestly predictive for ^1^ *D* (adjusted *R* ^2^ = .29; Table 3), and a plot of predicted ^1^ *D* as a function of both Disturbance and NEP (Figure 3a; to match Figure 1c–h) was most consistent with the hypothetical Figure 1f,g. Moreover, both productivity and disturbance clearly had quadratic effects on ^1^ *D* (Table 3; Figure 3b,c). We concluded that the DEM was supported for the diversity of the aquatic vegetation in the mesocosms.

During the year‐long outdoor experiment, one mesocosm leaked and did not maintain water depth; it was omitted from all analyses above. Surprisingly, only two other mesocosms became dominated by dense phytoplankton that interfered with the visual assessment of percent cover (i.e., the basis for ^1^ *D* results above). Phytoplankton blooms resulted in turbidity that reduced light at mesocosm substrates and potentially caused sparse growth of benthic macrophytes. Fortunately, production was directly measured throughout the study and biomass‐based analyses for macrophytes remained possible for those two mesocosms. Accordingly, plant biomass results are based on 39 mesocosms, whereas percent cover results are based on 37 mesocosms. Surprisingly, only five macrophyte species were observed during the 1‐year experiment: two macroalgae (*Chara*, *Nitella*) and three vascular plants (*Hydrilla verticulata*, *Najas guadalupensis*, and *Vallisneria americana*). In addition, filamentous algae were prominent in some mesocosms.

As a result of the relatively simple communities, taxon richness (^0^ *D*) was constrained in its range and response to treatments: the null model was most plausible (AIC_c_ *w*_*i*_ = 0.45; next δAIC_c_ = 2.2). By contrast, Pielou's evenness *J*' = (i.e., *H*′/*H*'_max_) detected a clear ∩ − shaped IPH effect, without a clear effect of disturbance (AIC_c_ *w*_*i*_ = 0.63; next δAIC_c_ = 2.4; *R* ^2^ = .27). We emphasized ^1^ *D* over *J*' because ^1^ *D* is unbounded whereas *J*' ranges from 0 to 1 and was more strongly constrained by limited richness.

Two dominant taxa help to understand community‐level effects for effective diversity (^1^ *D*). The macroalga *Chara* was most prevalent among macrophytes in terms of the mean percent cover and biomass. *Chara* fragments (as via physical disturbance here) can grow, and the most plausible model for *Chara* mean percent cover (modeled with a GLM using beta‐inflated distribution) included only a weak but positive effect of disturbance (effect = 0.15; *p* = .07; Nagelkerke *R* ^2^ = .09). By contrast, the mean percent cover of filamentous algae responded positively to production (effect = 0.26; *p* = .06) and negatively to disturbance (effect = −0.22; *p* = .10), though “explained” variation was modest (Nagelkerke *R* ^2^ = .14).

Whereas percent cover at three depths represented living structure in mesocosms, dry biomass consolidated macrophyte tissue to a single value per taxon per mesocosm. Perhaps due to that simplicity, biomass results did not detect effects of disturbance, with the most plausible model (AIC *w*_*i*_ = 0.38) supporting only a nonsignificant effect of NEP (*p* = .7) that could not be distinguished from the null (δAIC = 0.6). We concluded that diversity effects of disturbance and NEP were more sensitively detected in the living vertical structure represented by percent cover estimates of macrophytes and filamentous algae than in consolidated dry mass of macrophytes only. Moreover, this result illustrated the value in using a variety of diversity measures in studies like this.

## 4 DISCUSSION

Our year‐long experimental test of the DEM (Huston, 2014) in outdoor mesocosms generated quantitative disturbance and productivity gradients to evaluate the effects of both treatments on local diversity. According to the DEM, disturbance and productivity should lead to a peaked local diversity contour (e.g., Figure 1d; Huston, 2014), though we reasoned that alternative shapes and orientations of that “hill” may also be possible (Figure 1e–g), depending on relative strengths of the two drivers and the interaction. We observed one such alternative for effective diversity; simpler diversity measures failed to find an effect, and we think we understand why.

More importantly, our results support and advance the >40‐year‐old DEM and its components (IDH and IPH), which have attracted debates (Adler et al., 2011; Fox, 2013; Huston, 2014; Mackey & Currie, 2001). With the advantage of hindsight, we suggest that the hypotheses are sound but that problems of evidence have hindered progress, including insufficient sample size; too few planned and well‐replicated experiments; categorical and indirectly measured disturbance or production instead of directly measured gradients; overemphasis on species richness as the sole measure of diversity, and relatively simple statistical tools by modern standards (Adler et al., 2011; Bolker, 2008; Burnham & Anderson, 2002; Groendahl & Fink, 2017; Janousek & Dreitz, 2020; Jenkins, 2015; Jenkins & Quintana‐Ascencio, 2020; Jost, 2006; Kneitel & Chase, 2004; Korpinen et al., 2007; Laliberté et al., 2013; Mackey & Currie, 2001; Pollock et al., 1998; Rigby & Stasinopoulos, 2005; Scholes et al., 2005; Svensson et al., 2007; Wilkinson, 1999). Accordingly, we infer that much of what is collectively understood about the IDH, IPH, and DEM must be reexamined to ensure valid conclusions are drawn.

We know of two experiments that have tested the DEM at local scales; other studies have used landscape scales. Our 1‐year experiment used disturbance and production gradients with replicated outdoor aquatic mesocosms and found that diversity obtained dual hump‐shaped responses, where production caused greater response than disturbance. Our experiment was intermediate in realism between an experiment using flasks in a growth chamber (Groendahl & Fink, 2017) and others in natural landscapes. Our study and that of Groendahl and Fink (2017) shared two important features for testing the DEM: (a) experimental designs with disturbance and production across wide ranges (to permit a full hump‐shaped response), and (b) a flexible set of expectations for a response surface given local disturbance and productivity effects (e.g., Figure 1d–g above). Here, physical disturbance and production were independent in a factorial experimental design that used biotic diversity as a response. Landscape‐scale studies (e.g., Chillo et al., 2020; Janousek & Dreitz, 2020; Laliberté et al., 2013) necessarily have greater difficulty generating independent disturbance and productivity treatments, and often use biological “disturbances” such as natural (coevolved) predators. There is clearly room for more strong experiments to test these important ideas well in varied study systems, where the challenge will be applying independent productivity and disturbance treatments.

Life histories of dominant organisms in our mesocosms help to understand the results here and relate to the assumed colonization‐competition tradeoff. The macroalga *Chara* increased by fragmentation with disturbance, consistent with vegetative growth via modular clonal anatomy and colonization abilities (Bociąg & Rekowska, 2012; Skurzyński & Bociąg, 2011). That fragmentation and growth enabled *Chara* to be resilient and thus mitigated overall disturbance effects. By contrast, filamentous algae decreased with disturbance but increased with production, where it out‐competed other organisms if the environment was stable and resources plentiful. *Chara* and *Hydrilla* each reproduce vegetatively and sexually, leading to substantial numbers of propagules that are resilient to disturbance (Langeland, 1996; Van den Berg et al., 2001). Thus production had a greater effect in determining the composition of the mesocosms than did disturbance, and a different set of species may have obtained a different outcome. The general DEM framework outlined here (Figure 1) permits variation in responses by different communities. Sufficient experimental design and knowledge of included taxa will help to more fully test the DEM across different systems.

Interestingly, effective diversity (based on percent cover at three depths) captured disturbance and production responses, while simpler biomass and richness did not. We infer that details of living plant structure were important here but missed when data were collapsed to simpler richness and biomass values for the relatively simple communities here. To the extent possible, it may be helpful to apply a similar 3D community composition approach to other experimental systems when testing for DEM effects, much like evaluations of forests (e.g., Guitet et al., 2018; Onaindia et al., 2004).

The use of outdoor mesocosms here made an intentional tradeoff between control of experimental conditions and realism. The mesocosm scale afforded the advantage of experimental design and replication, making for a robust test of the DEM at local scales through a year's colonization and growth. The corresponding disadvantage was that uncontrolled outside factors (e.g., succession) were also at play in the outdoor mesocosms during the year‐long experiment. Accordingly, treatments represented ~20% of variance though disturbance treatments ranged from 0% to 96% of benthic surface raked repeatedly and cumulative NEP values covered a twofold range. Experiments designed similarly, but in other, more diverse settings are needed to more fully evaluate the DEM and its components.

Our results support the DEM, enable a broader basis for its expectations (Figure 1d–g), and point toward more robust tests of the DEM and its components (IDH and IPH). The effects of disturbance *and* productivity on biological diversity remain shrouded by a convoluted history of scientific inquiry. Our methods and results may advance toward a better understanding of these important concepts.

## AUTHOR CONTRIBUTIONS

**Jacob D. Hart:** Data curation (equal); formal analysis (equal); investigation (equal); methodology (equal); project administration (equal); visualization (equal); writing – review and editing (equal). **David G. Jenkins:** Conceptualization (lead); data curation (equal); formal analysis (equal); investigation (equal); methodology (equal); project administration (equal); visualization (equal); writing – original draft (equal); writing – review and editing (equal).

## FUNDING INFORMATION

This experiment was conducted without external funding support.

## CONFLICT OF INTEREST STATEMENT

The authors declare no competing interests.

## Data Availability

Data collected and analyzed for this manuscript are provided at: https://doi.org/10.5061/dryad.jsxksn0b3. Novel code for analyses and figures presented here is linked to Dryad from Zenodo and may be accessed at the same URL.
